# The Oncogenic Role of UBXN1 in Gastric Cancer Is Attributed to the METTL16‐Mediated m6A Methylation and Histone Modifications

**DOI:** 10.1002/cam4.70772

**Published:** 2025-03-17

**Authors:** Kesong Shi, Yani Chen, Tian Gao, Hua Guo, Xinyao Fu, Yuan Wu, Haiquan Yu

**Affiliations:** ^1^ The State Key Laboratory of Reproductive Regulation and Breeding of Grassland Livestock Inner Mongolia University Hohhot China

**Keywords:** gastric cancer, histone modification, m6A modification, METTL16, UBXN1

## Abstract

**Background:**

Multiple epigenetic regulatory mechanisms are crucial in tumorigenesis and development. However, the synergistic relationship between N6‐methyladenosine (m6A) and histone modifications in regulating gene expression of gastric cancer (GC) requires further investigation.

**Results:**

Here, based on the microarray, RNA‐seq, and survival analysis data, the m6A methyltransferase METTL16 was identified as a potential tumorigenic factor of GC. The silence of METTL16 suppresses the malignant phenotype of GC cells, and the NF‐κB pathway was activated. By using the weighted correlation network analysis (WGCNA) and integrating RNA‐seq and MeRIP‐seq data, it was found that METTL16 is significantly positively correlated with UBX domain protein 1 (UBXN1). Furthermore, through the MeRIP‐qPCR and dual‐luciferase reporter assays, we found that knocking down METTL16 reduced the m6A modification level of the UBXN1 coding sequence in GC. Interestingly, the silencing of METTL16 also downregulated UBXN1 expression by promoting H3K36me3 modification at the UBXN1 promoter. Subsequent investigations found that the silencing of METTL16 upregulated the expression of the major H3K36me3 methyltransferase SETD2 in GC cells by methylating the m6A site in the mRNA coding sequence of SETD2.

**Conclusions:**

Our findings demonstrate the spatio‐temporal regulation of UBXN1 expression in GC cells by METTL16 through a combination of transcriptional and post‐transcriptional mechanisms. The synergistic interplay of these various epigenetic mechanisms provides new prospects for tumor diagnosis and precision treatment.

## Graphical abstract

In this study, we demonstrated that METL16 is essential for GC progression by connecting m6A modification with histone modifications. The regulatory role of METTL16, which suppresses the malignant phenotype of GC cells at both the transcriptional and post‐transcriptional levels, offers new prospects for tumor diagnosis and precision treatment.

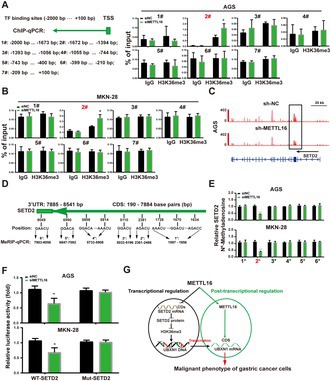

**Abbreviations**
AUC: Area under the curve
CDS: Coding sequences
GC: Gastric cancer
m6A: N6‐methyladenosine
MeRIP‐seq: Methylated RNA immunoprecipitation sequencing
METTL16: Methyltransferase‐like 16
ncRNAs: Non‐coding RNAs
ROC: Receiver operating characteristic
siRNAs: Small interfering RNAs
UBXN1: UBX domain protein 1
WGCNA: Weighted correlation network analysis

## 1 Introduction

Gastric cancer is one of the most common malignant tumors in the world. The systemic treatment of gastric cancer, including chemotherapy, targeted therapy, and immunotherapy, has made significant advancements in recent years [1]. However, the prognosis for GC patients remains unfavorable [2]. The potential impact of epigenetic regulatory mechanisms at the transcription and post‐transcription levels on cancer development has attracted increasing research attention [3, 4]. Consequently, further investigations into the interplay of these epigenetic regulatory mechanisms may provide valuable perspectives for GC prevention.

The development of GC is subject to epigenetic regulation at the transcription, post‐transcription, and translation levels [5]. Among them, non‐coding RNAs (ncRNAs) [6], DNA methylation [7], and histone modifications [8] have been widely investigated. RNA modification, as an epigenetic regulator, has recently attracted increasing attention [9]. Among the various RNA modifications, m6A RNA modification has become an important regulatory factor in the spatio‐temporal gene expression during cancer development [10, 11]. These processes are dynamic and reversible, facilitated by methyltransferases and demethylases [12]. The m6A “readers” selectively bind to methylated mRNA and control RNA destiny in a methylation‐dependent way, governing mRNA stability, degradation, and translation [13]. A growing body of evidence has indicated that m6A‐related enzymes are involved in the carcinogenesis of various malignancies [12, 14, 15]. Additionally, m6A and other epigenetic modifications have been shown to demonstrate synergistic effects [16, 17, 18]. The m6A modification regulates the production, stability, and degradation of ncRNAs, and conversely, ncRNAs play a role in modulating the expression of proteins associated with m6A [19, 20]. In previous research, METTL3, the m6A methyltransferase, cooperates to produce synergistic impacts on m6A methylation and histone alterations, thereby regulating VGF function in lung adenocarcinoma [21]. However, the interplay between m6A and histone modification in the progression of GC remains unclear.

In this study, utilizing multi‐omics data, we identified m6A‐related genes related to GC prognosis, including METTL16 and its targeted molecules. Furthermore, we investigated their regulatory effects on target genes and the combined influences of various epigenetic mechanisms.

## 2 Materials and Methods

### 2.1 Data Sources and Bioinformatics Analysis

The MeRIP‐seq, RNA‐seq, and microarray data were obtained from the GEO database (http://www.ncbi.nlm.nih.gov/geo). The details are shown in Table S1. Detailed data processing procedures for MeRIP‐seq, RNA‐seq, and microarray data were outlined in our prior studies [21, 22]. For MeRIP‐seq data, the m6A peak locations were identified and visualized using the Guitar package (v2.8.0) [23]. In the context of WGCNA, soft‐threshold parameters with a scale‐free R^2^ = 0.90 were selected. Complete code for WGCNA analyses is included in the Supporting Table. Survival analysis was performed with Kaplan–Meier Plotter. Open the Kaplan–Meier Plotter database website (http://www.kmplot.com, 2023 version), select the mRNA (gene chip) gastric cancer dataset from the all‐cancer types database, and enter the gene symbol. Then, select overall survival (OS) and set the median as the cutoff value. Keep all other parameters as default. Click “Draw Kaplan‐Meier plot” to generate the logrank *p* value and hazard ratio (HR). Finally, use ggplot2 to visualize the results as a forest plot.

### 2.2 Gastric Cancer Samples

The cDNA microarray (cDNAHStmA030CS01) used in this study was acquired from Shanghai Outdo Biotech Company in Shanghai, China. It consists of 15 GC tissues and 15 adjacent noncancerous tissues. Furthermore, an additional set of 55 GC tissues and 55 adjacent noncancerous tissues (IWLT‐C‐110G12) was obtained from the same company. Ethical approval for this study involving human participants was granted by the Ethics Committee of Shanghai Outdo Biotech Company (approval number: YB M‐05‐02).

### 2.3 Cell Culture, Gene Overexpression, and Knockdown

The human GC cell line (AGS, MKN‐28) and human normal gastric epithelial cell GES‐1 were cultured in DMEM/F12 and DMEM (VivaCell, Shanghai, China) supplemented with 10% fetal bovine serum (Gibco, Waltham, MA, USA), respectively. The complete CDS of human UBXN1 mRNA was amplified and cloned into the pIRES2‐ZsGreen1 vector (Clontech, Takara, Tokyo, Japan) to generate the overexpression plasmid. The small interfering RNAs (siRNAs) targeting METTL16 or UBXN1 were custom‐designed and synthesized by Genepharma in Shanghai, China. Specific sequences of the siRNAs can be found in Table S2.

### 2.4 qRT–PCR and Western Blot Analysis

Total RNA was extracted with TRIzol reagent (Invitrogen, Carlsbad, CA, USA), followed by qRT‐PCR analysis conducted on an ABI 7500 instrument (Applied Biosystems, Foster, USA) using GoTaq qPCR Master Mix (Promega, Wisconsin, USA). The primer sequences can be found in Table S3. The primers were synthesized by Huada Gene Company (Beijing, China).

Cells were lysed with RIPA lysis buffer supplemented with protease inhibitors (R0010, Solarbio, Beijing, China). The extracted proteins were subsequently separated and transferred onto PVDF (0.45 μm) membranes (Millipore, Bedford, MA, USA). Following blocking with non‐fat milk (Hercules, CA, USA), the membranes were incubated with primary antibodies overnight at 4 °C. Subsequently, the membranes were washed and exposed to secondary antibodies for 1 h at room temperature the next day. Table S4 contains the antibody details, while the western blot raw data includes the original images.

### 2.5 Cell Proliferation Assay

For the CCK‐8 assay, AGS and MKN‐28 cells that were transfected were plated at a density of 1 × 10^3^ cells per well on 96‐well plates (Corning, New York, USA) and then treated with 10 μL of CCK‐8 (TransGen, Beijing, China) reagent. Cell counts were performed every 24 h for three days. The EdU incorporation assay was utilized to estimate cell viability, and it was conducted according to the manufacturer's instructions using EdU reagent (C0075s, Beyotime, Shanghai, China).

### 2.6 Cell Invasion and Migration

A total of 2 × 10^5^ cells were seeded in transwell plates (Corning, New York, USA), with some being coated with Matrigel (Corning, MA, USA) and others left uncoated. The cells were treated with 4% paraformaldehyde (Solarbio, Beijing, China) for 30 min and subsequently stained with 0.1% crystal violet (C0121, Solarbio, Beijing, China). Furthermore, for the scratch assay, cells were plated in 6‐well plates (Corning, New York, USA) at a density of 2 × 10^5^ cells per well. Upon reaching full confluence, a 10 μL sterile pipette tip (Kirgen, Shanghai, China) was used to create an artificial scratch. The migration ability of the cells was then assessed by determining the percentage of wound‐healing rate at 24 h and 48 h after the scratch was made.

### 2.7 RNA Stability

Following gene manipulation, cells were exposed to 10 μg/mL of actinomycin D (Sigma‐Aldrich, Munich, Germany) in the cell medium. Subsequently, cells were collected at time points of 0, 4, 8, and 12 h to extract RNA. The expression levels of SETD2 mRNA were determined via qRT–PCR using the primers detailed in Table S3.

### 2.8 MeRIP‐qPCR Assay

The MeRIP assay was performed using the MeRIP Kit (EPIGENTEK, New York, USA). The total RNA was fragmented into 100–200 nucleotides and then underwent RNA immunoprecipitation with m6A antibody for subsequent qRT‐PCR analysis. Refer to Table S5 for the primer sequences.

### 2.9 Dual‐Luciferase Reporter Assay

Fragments of UBXN1‐CDS/SETD2‐CDS containing both wild‐type and mutant m6A motifs were directly synthesized by Beyotime (Shanghai, China). These fragments were subsequently inserted into the luciferase reporter vector pmirGLO (Promega, Madison, USA). Cells were then seeded into individual wells of a 6‐well plate (Corning, New York, USA) and co‐transfected with the vectors following the JetPRIME reagent protocol (Polyplus, Illkirch, France). Luciferase activity was determined using the dual‐luciferase reporter assay system (Promega, Wisconsin, USA).

### 2.10 ChIP Assays

The ChIP assays were conducted using the Simple ChIP Enzymatic Chromatin IP Kits (Cell Signaling Technology, MA, USA). Quantification of the immunoprecipitated DNA was carried out using qRT–PCR with SYBR Green Mix (Promega, Wisconsin, USA). Table S6 contains the primer sequences for the UBXN1 promoter.

### 2.11 In Vivo Tumor Formation Assay

AGS cells (1 × 10^7^) transfected with the specified material were combined with 50% (v/v) Matrigel (Corning, MA, USA) and subsequently subcutaneously injected into both the left and right dorsal areas of female BALBc nude mice (SPF, Beijing, China). The AGS cells (1 × 10^7^) was subcutaneously injected weekly. One month following transplantation, the mice were euthanized. Based on the guidance from the American College of Laboratory Animal Medicine, CO2 euthanasia has been selected as a humane method. Thus, this study utilized CO2 euthanasia for mouse euthanasia. The procedure involves the gradual introduction of 100% CO2 (NEWRADAR, Wuhan, China) at a flow rate of 4.6 L/min, with the gas flow sustained for a minimum of 1 min after the apparent death of the animal. Tumor volume was determined using the formula 1/2 × length × width^2. Female BALB/c nude mice were selected and randomly grouped, with six mice per group. However, in every group, two mice did not develop tumors.

### 2.12 Statistical Analysis

The data was analyzed using Prism Graphpad 8.0 software (San Diego, CA, USA) and is presented as the mean ± standard deviation. Each experiment was replicated three times unless otherwise stated. Significant differences were assessed using a two‐tailed Student's *t*‐test or one‐way ANOVA for comparisons between multiple groups.

## 3 Results

### 3.1 Screening of Key m6A Regulators in Gastric Cancer

**FIGURE 1:**
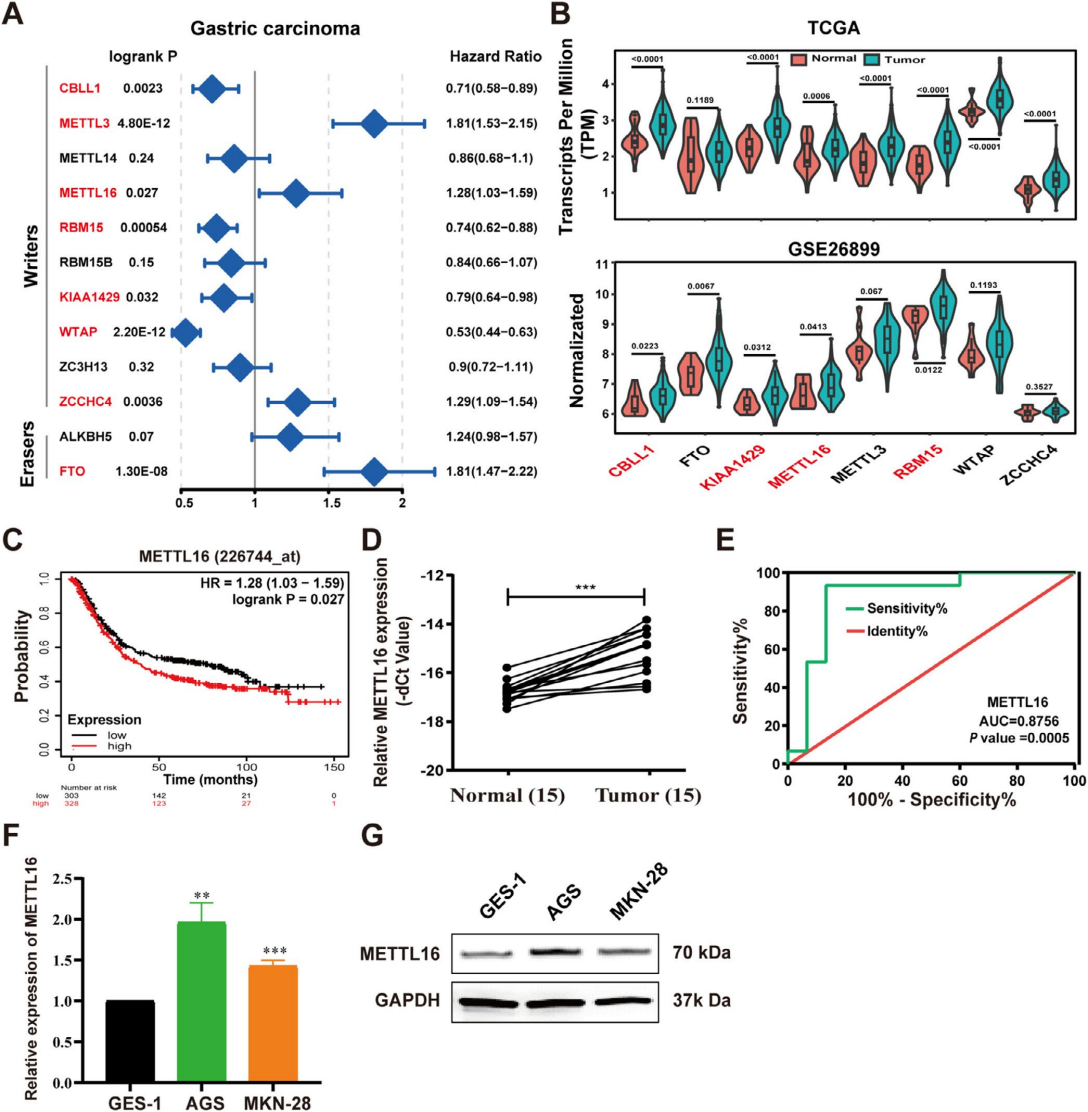
Screening of key m6A regulators in gastric cancer. (A) Forest map of m6A regulators on survival analysis in GC. (B) *METTL16* expression in GC tissues and adjacent normal tissues from the TCGA and GEO datasets. (C) Prognostic analyses for GC patients. (D) *METTL16* expression levels in GC tissues and adjacent normal tissues were detected by qRT‐PCR. (E) ROC curve analysis of the *METTL16* gene. (F–G) qRT‐PCR and western blot verification of METTL16 expression. Bar = mean ± SD (*n* = 3). **p < 0.01, ***p < 0.001.

To screen key m6A regulators in GC. Firstly, we assessed the impact of m6A regulators (methyltransferases and demethylases) on survival probability using the KM plotter tool (https://kmplot.com/). The results showed that there is a significant correlation between the expression of *CBLL1*, *METTL3*, *METTL16*, *RBM15*, *KIAA1429*, *WTAP*, *ZCCHC4*, and *FTO* and the prognosis of GC (*logrank p <* 0.05) as illustrated in Figure 1A. Subsequent investigations demonstrated that *CBLL1*, *KIAA1429*, *METTL16*, and *RBM15* were highly expressed in GC tissues (Figure 1B). However, the gene expression and prognosis of *CBLL1*, *KIAA1429*, and *RBM15* are not consistent; the high expression of *CBLL1*, *KIAA1429*, and *RBM15* led to better prognosis, while the high expression of *METTL16* led to poor prognosis of GC patients (Figure 1A,C). Therefore, METTL16 was selected as the subject for the subsequent experiments. According to qRT‐PCR analysis, METTL16 exhibited upregulation in GC tissues (Figure 1D). Additionally, the diagnostic potential of METTL16 as a biomarker for GC was evaluated through a receiver operating characteristic (ROC) curve. The area under the curve (AUC) was calculated to be 0.8756 (Figure 1E). Furthermore, there was significant upregulation of METTL16 in GC cells (AGS and MKN‐28) compared to GES‐1 cells (Figure 1F–1G). These results imply that METTL16 may be involved in regulating the progression of GC.

### 3.2 METTL16 Knockdown Inhibits the Malignant Phenotype of GC Cells

**FIGURE 2:**
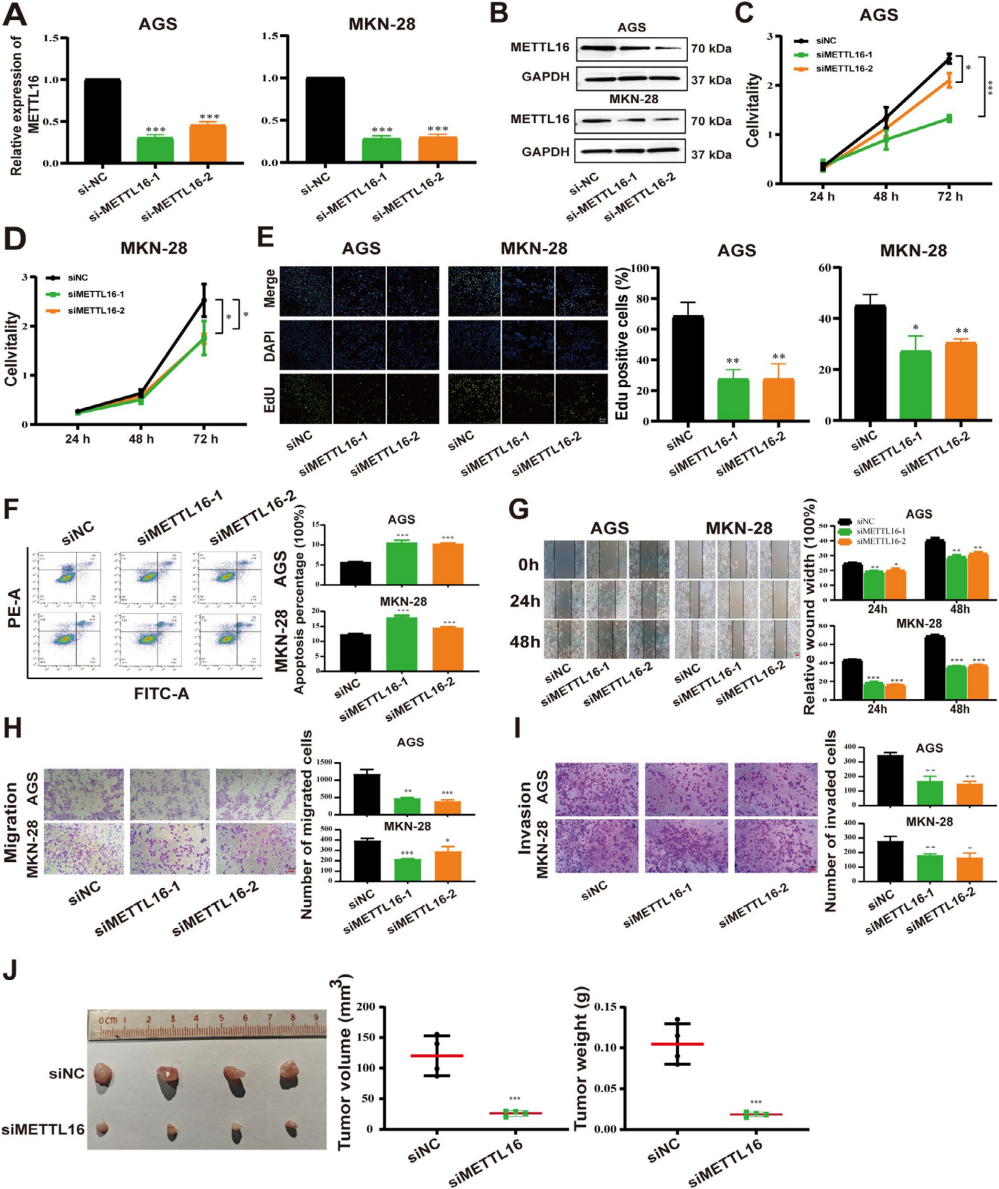
METTL16 knockdown inhibits proliferation, migration, and invasion of GC cells. (A, B) qRT‐PCR and western blot analysis of METTL16 knockdown efficiency in AGS and MKN‐28 cells. (C, E) Cell proliferation viability was analyzed by CCK‐8 and EdU. (F) Effect of METTL16 knockdown on cell apoptosis in GC cells. (G, I) Migration and invasion assays for AGS and MKN‐28 cells. (J) The effect of METTL16 knockdown on AGS cells subcutaneous xenografts in vivo. Bar = mean ± SD (*n* = 3). *p < 0.05, **p < 0.01, ***p < 0.001.

We next investigated the effects of METTL16 knockdown on proliferation, migration, and invasion of GC (AGS, MKN‐28) cells. According to the qRT‐PCR and western blot assay results, the expression of METTL16 was significantly downregulated in GC cells transfected with siRNA targeting METTL16 (siMETTL16) (Figure 2A–2B). Additionally, METTL16 knockdown notably suppressed the proliferation of AGS and MKN‐28 cells (Figure 2C–2E). Flow cytometric analysis revealed that METTL16 knockdown increased apoptosis in GC cells (Figure 2F). The results of wound healing and transwell assays indicated reduced cell migration and invasion upon METTL16 knockdown (Figure 2G–2I). The METTL16 knockdown groups demonstrated reduced tumor size and slower tumor growth in vivo experiments compared to the control group (Figure 2J).

### 3.3 METTL16 Positively Regulates UBXN1 mRNA via m6A Modification

**FIGURE 3:**
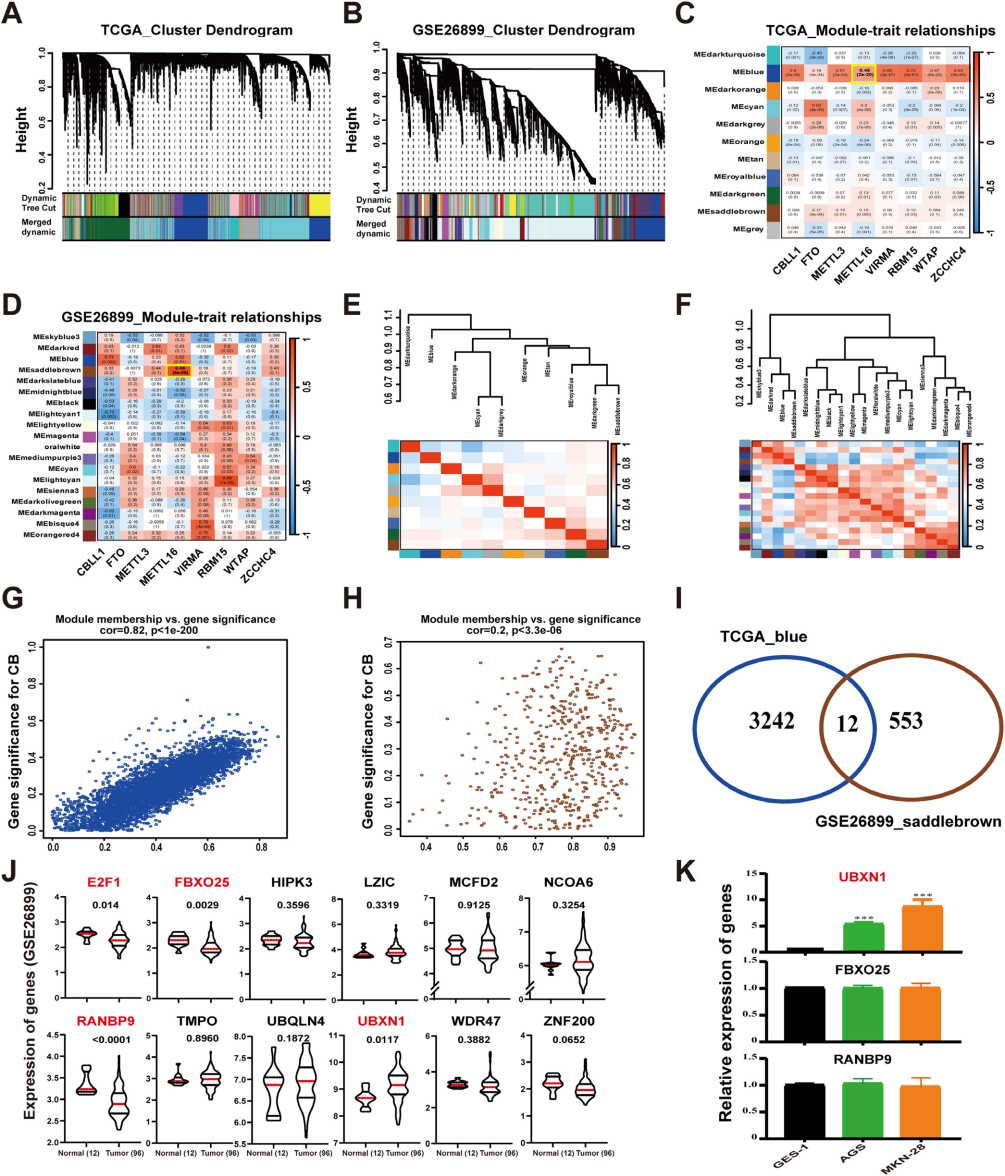
WGCNA of the m6A regulators genes. (A, B) Hierarchical clustering tree. (C, D) The correlation between the gene module and prognosis‐related m6A regulators. (E, F) Clustering module hub genes by hierarchical structure (top) and heatmap of the adjacencies in the hub gene network (below). (G, H) Scatter plot of the GS for the grade vs. the MM in the blue and saddlebrown module. (I) Venn diagram illustrating the shared number of genes. (J) Gene expression in GC samples. (K) UBXN1 mRNA expression patterns in GC cells. Bar = mean ± SD (*n* = 3). ***p < 0.001.

**FIGURE 4:**
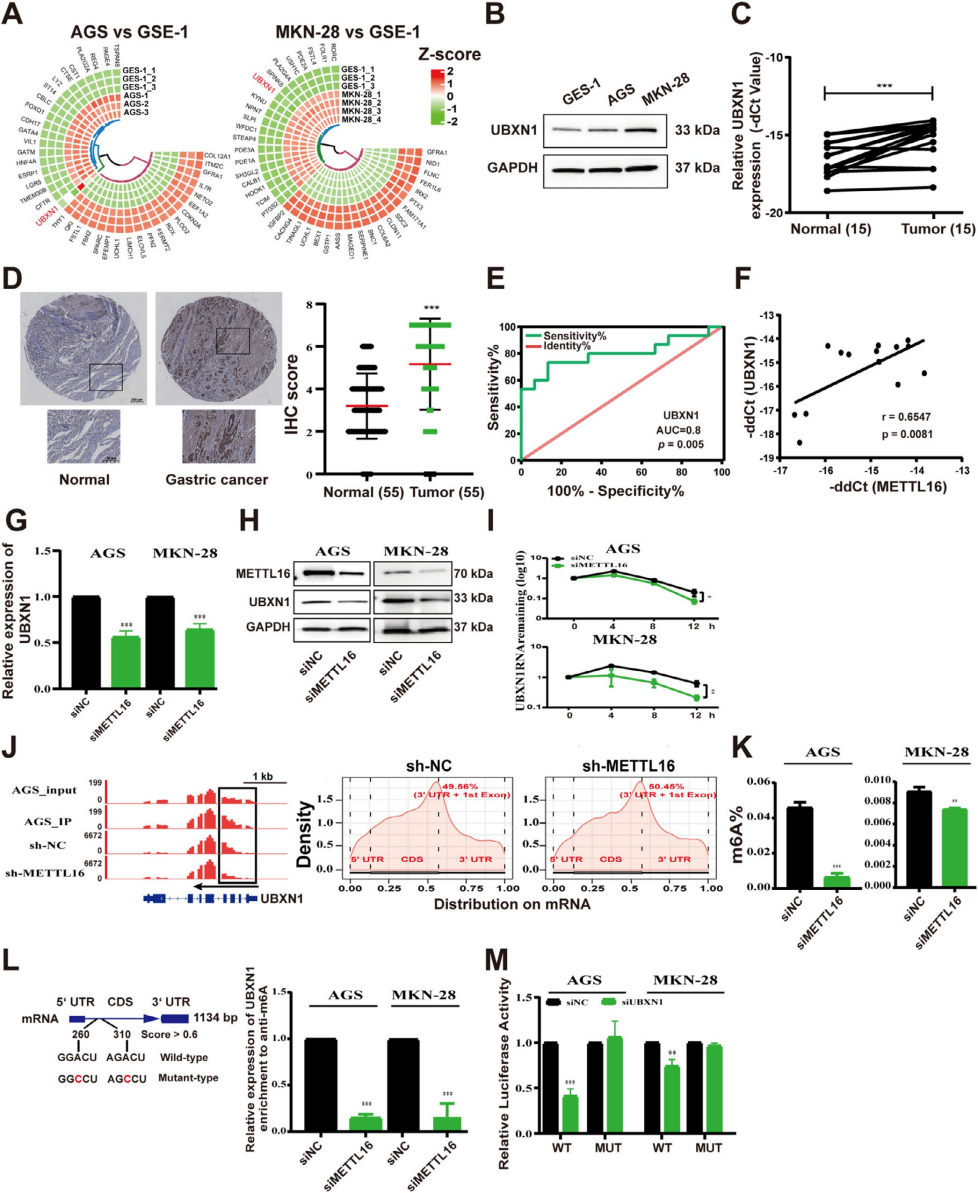
METTL16 positively regulates UBXN1 mRNA via m6A modification. (A) Differentially expressed genes. (B, D) UBXN1 mRNA expression and protein expression patterns in GC cells and GC tissues. (E) ROC curve analysis of the *UBXN1* gene. (F) METTL16 and UBXN1 correlation in GC tissues and adjacent normal tissues. (G, H) qRT‐PCR and western blot analysis of the expression of UBXN1 with METTL16 knockdown in AGS and MKN‐28 cells. (I) UBXN1 mRNA was analyzed in AGS and MKN‐28 cells after actinomycin D treatment. (J) MeRIP‐seq read distributions in UBXN1 (left) and the mRNA density coverage (right). (K) The total m6A level of AGS and MKN‐28 cells. (L, M) Schematic photo of CDS‐WT, CDS‐mutant in *UBXN1* mRNA, MeRIP‐qPCR, and dual‐luciferase assay results. Bar = mean ± SD (*n* = 3). *p < 0.05, **p < 0.01, ***p < 0.001.

METTL16 has been investigated for its role in regulating cancer progression by modulating target genes through m6A modification [24, 25, 26]. Hence, additional analysis was conducted to uncover the target genes influenced by METTL16 through m6A modification in GC. WGCNA analysis was executed utilizing prognosis‐associated m6A regulators and genes from the TCGA‐STAD and GSE26899 datasets. The co‐expression modules were established from the scale‐free network and displayed using dynamic tree cutting (Figure 3A–3B). In the TCGA‐STAD and GSE26899 datasets, 11 and 19 modules were respectively identified (Figure 3C–3D). Figure 3E–3F showed that the modules were independent of each other. Notably, the blue and saddlebrown modules exhibited a significant positive correlation with METTL16 in TCGA‐STAD and GSE26899, respectively (*r* > 0.4, *p value* < 0.01) (Figure 3C–3D). Furthermore, the correlation was found to be significant in the blue (*r* = 0.46, *p value* = 2e‐20) and saddlebrown (*r* = 0.85, *p value* = 6e‐05) modules, respectively (Figure 3G–3H). A total of 12 overlapping genes in the blue (TCGA‐STAD) and saddlebrown (GSE26899) modules were identified (Figure 3I). Among them, *E2F1*, *FBXO25*, *RANBP9*, and *UBXN1* were differentially expressed (*p value* < 0.05) in between GC tissues and normal tissues and were associated with prognosis in patients with GC (Table S7; Figure 3J). However, the gene expression and prognosis of *E2F1* are not consistent; the low expression of *E2F1* led to better prognosis (Table S7; Figure 3J). Further research found that only UBXN1 was highly expressed in GC cell lines (AGS, MKN‐28) relative to GES‐1 cells (Figure 3K). Moreover, the expression pattern of UBXN1 in AGS, MKN‐28, and GES‐1 is analyzed using RNA‐seq data available in the GEO database. Compared with that in GES‐1 cells, UBXN1 in AGS and MKN‐28 cells was significantly upregulated (Figure 4A). This finding was further confirmed by western blot analyses (Figure 4B). According to qRT‐PCR (Figure 4C) and immunohistochemistry analyses (Figure 4D), UBXN1 was significantly elevated expression in GC tissues. Furthermore, the AUC was calculated to be 0.80 (Figure 4E). An examination of the correlation between UBXN1 and METTL16 gene expression revealed a positive association in GC tissues (*r* = 0.6547, *p value* = 0.0081) (Figure 4F). Subsequently, qRT‐PCR (Figure 4G) and western blot (Figure 4H) analyses demonstrated a decrease in the expression of UBXN1 in GC cells following METTL16 silencing. Furthermore, the impact of METTL16 knockdown on the stability of UBXN1 mRNA was investigated, revealing a decrease in UBXN1 mRNA stability in AGS and MKN‐28 cells (Figure 4I).

Based on the published MeRIP‐seq data (GSE224890) of AGS cells, we detected higher m6A occupancy of UBXN1 in AGS IP samples compared to AGS input samples, and a decreased m6A occupancy of UBXN1 was observed in METTL16‐depleted AGS cells compared to control (Figure 4J). Moreover, the highest number of m6A peaks was identified in the stop codon and 3' UTR in both METTL16‐depleted (sh‐METTL16) and control (sh‐NC) AGS cells (Figure 4J). Consistently, knockdown of METTL16 consistently resulted in reduced overall m6A modification levels in AGS and MKN‐28 cells compared to the control (Figure 4K). Furthermore, the SRAMP online tool [27] unveiled two significant m6A sites in the CDS of UBXN1 mRNA (Figure 4L). MeRIP‐qPCR analysis revealed a notable decrease in m6A levels of fragments linked to the specific site upon METTL16 knockdown (Figure 4L), while dual‐luciferase reporter assays showed a reduction in luciferase activity with wild‐type UBXN1 but not with mutated UBXN1 following METTL16 knockdown (Figure 4M). These findings suggest that METTL16 regulates UBXN1 expression by methylating the m6A site in the CDS of UBXN1 mRNA in GC cells.

### 3.4 METTL16‐Mediated m6A mRNA Modification of UBXN1 mRNA Promotes GC Progression

**FIGURE 5:**
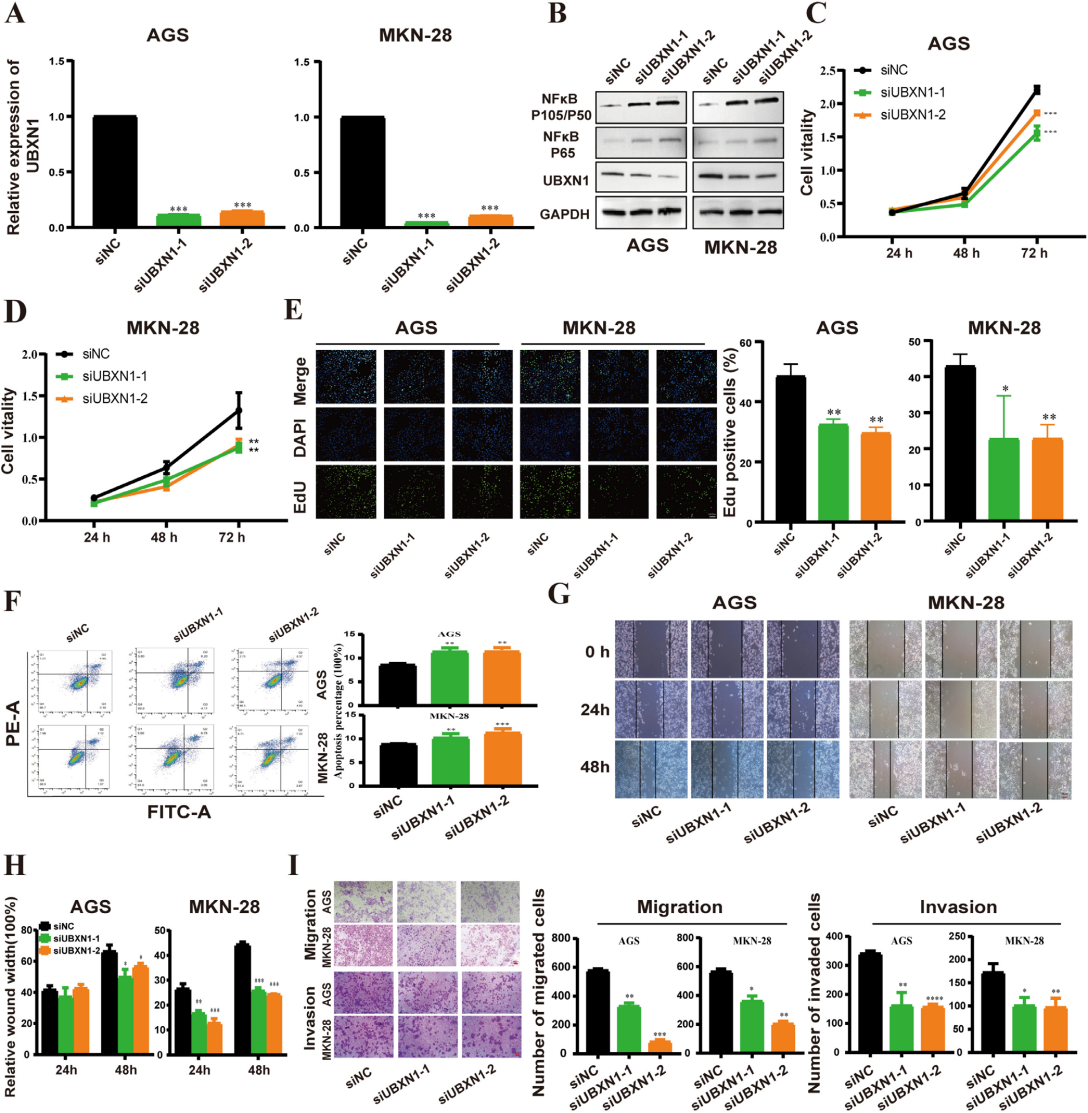
UBXN1 knockdown repressed the malignant phenotype of AGS and MKN‐28 cells in vitro. (A, B) The efficiency of UBXN1 knockdown was verified by qRT, PCR and western blot. (C, E) CCK8 and EdU assays were used to test the GC cell viability. (F) Flow cytometry analysis of apoptosis. (G)‐(I) Cell migratory and invasive abilities were detected. Bar = mean ± SD (*n* = 3). *p < 0.05, **p < 0.01, ***p < 0.001.

**FIGURE 6:**
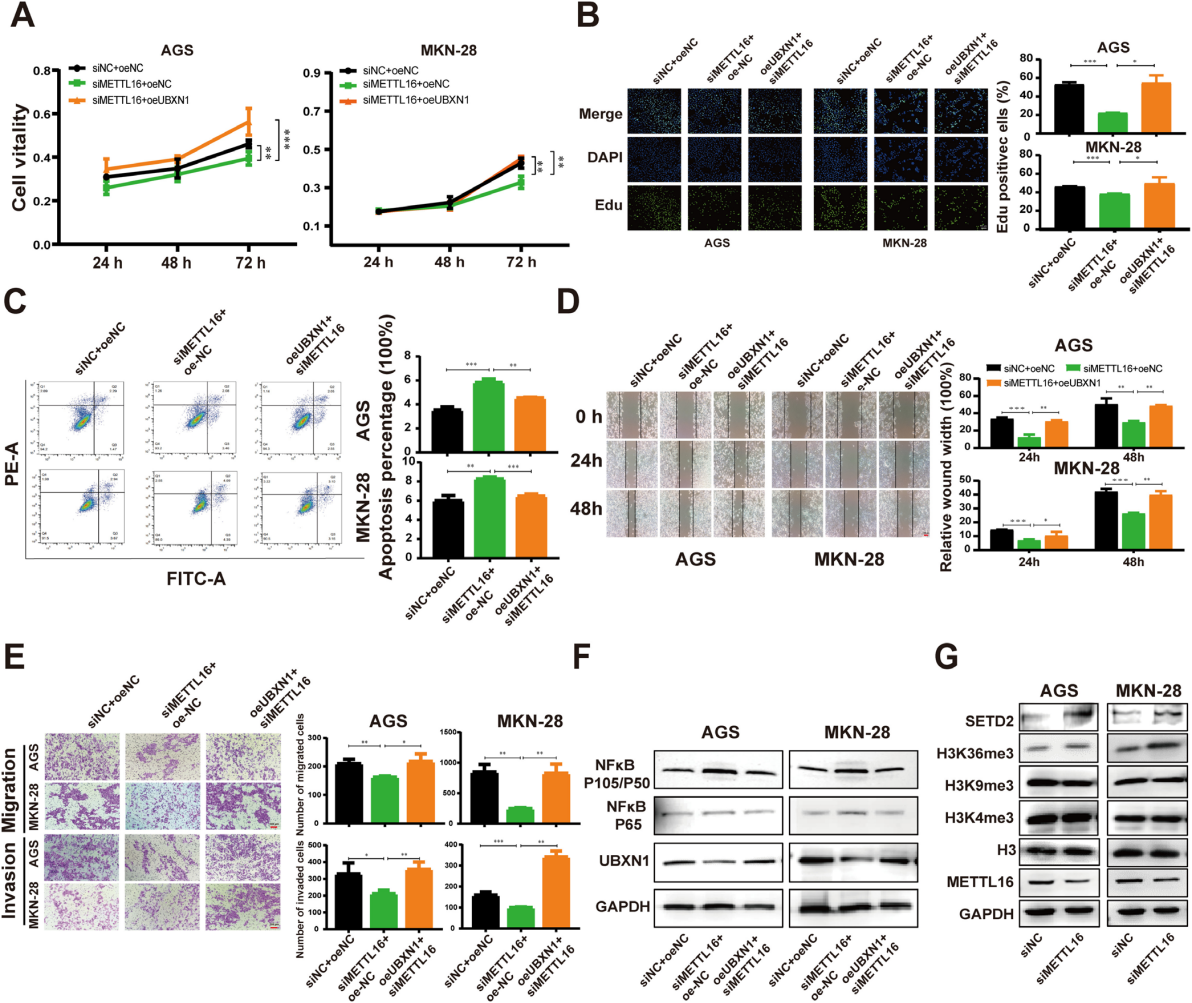
METTL16‐mediated m6A mRNA modification of UBXN1 mRNA promotes GC progression. (A, B) Cell viability of AGS and MKN‐28 was assessed using CCK8 and EdU assays. (C) Flow cytometry analysis of apoptosis in AGS and MKN‐28 cells. (D, E) Cell migratory and invasive abilities (F, G) western blot results of NF‐κB, SETD2, and H3 proteins. Bar = mean ± SD (*n* = 3). *p < 0.05, **p < 0.01, ***p < 0.001.

Given that METTL16 acts as a positive regulator of UBXN1 mRNA through m6A modification, a study was carried out to assess whether METTL16 facilitates the advancement of GC by controlling m6A modification of UBXN1 mRNA. GC cells (AGS and MKN‐28) were transfected with siNC or siUBXN1. Subsequent qRT‐PCR and western blot analyses revealed a significant decrease in UBXN1 expression following transfection with siRNA‐UBXN1 compared to controls (Figure 5A,5B). Functional analyses, including cell proliferation, cell migration, and invasion assays, demonstrated that UBXN1 knockdown inhibited the malignant phenotype of AGS and MKN‐28 cells in vitro (Figure 5C–5I), while UBXN1 overexpression exhibited the opposite effect (Figure S1A–H). In order to investigate the potential role of UBXN1 in modulating the NF‐κB signaling pathway in GC cells, the impact of UBXN1 knockdown on NF‐κB signaling was assessed via western blot analysis. The findings revealed that UBXN1 knockdown elevated the phosphorylation levels of NF‐κB (Figure 5B), whereas overexpression of UBXN1 led to the opposite outcome (Figure S1B). Rescue experiments found that UBXN1 overexpression largely reversed the inhibitory effect of the knockdown of METTL16 on AGS and MKN‐28 cell proliferation, migration, and invasion (Figure 6A–6E). In addition, the knockdown of METTL16 increased the phosphorylation level of NF‐κB, which was recovered by UBXN1 overexpression (Figure 6F).

### 3.5 METTL16 Modulates the H3K36me3 Level by Mediating m6A Modification of SETD2 mRNA

**FIGURE 7:**
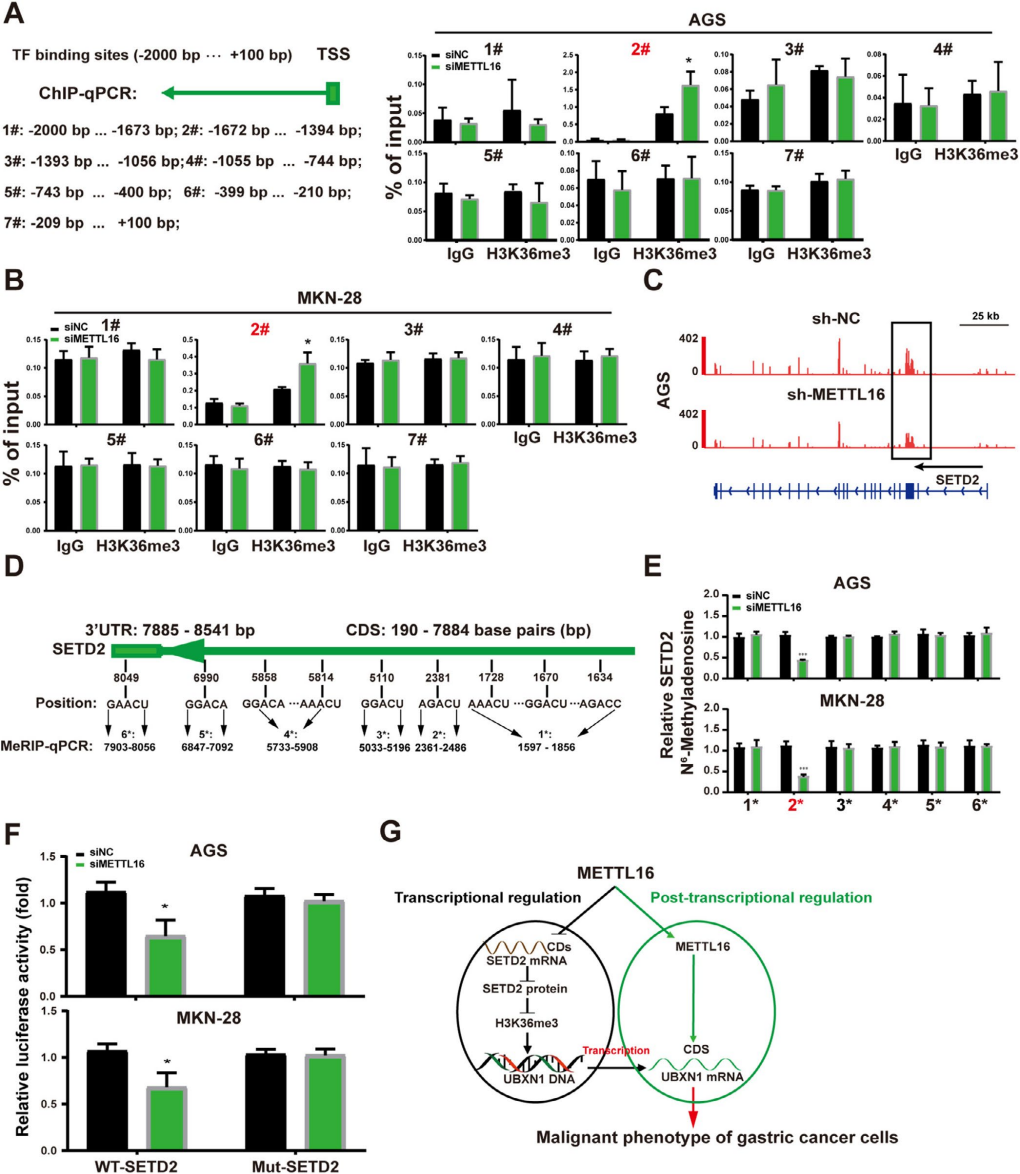
METTL16 modulates the H3K36me3 level by mediating m6A modification of SETD2 mRNA. (A, B) ChIP, qPCR analysis of H3K36me3 at UBXN1 in AGS and MKN‐28 cells. (C) The distribution of MeRIP‐seq reads in the SETD2 locus. (D) The CDS and 3'UTR sequences of *SETD2* mRNA were inspected for predicted m6A sites. (E) The m6A levels of m6A sites of *SETD2* mRNA in AGS and MKN‐28 cells were analyzed by MeRIP‐qPCR. (F) Dual‐luciferase assay result. (G) Schematic showing the functional and molecular mechanisms of METTL16 in GC cells. Bar = mean ± SD (*n* = 3). *p < 0.05, ***p < 0.001.

An assessment was conducted to investigate whether METTL16 is involved in the regulation of H3 tri‐methylation. The findings revealed that reducing METTL16 led to a significant rise in H3K36me3 levels, as opposed to other H3 proteins, when compared to the control group (Figure 6G). Moreover, ChIP–qPCR data exhibited that the reduction of METTL16 led to elevated H3K36me3 levels at the promoter region of UBXN1 in AGS and MKN‐28 cells (Figure 7A–7B). Figure 6G showed that the expression of SETD2 protein, the primary methyltransferase for H3K36me3, was notably increased in METTL16 knockdown AGS and MKN‐28 cells. Based on the MeRIP‐seq data from METTL16‐depleted AGS cells (GSE224890), a decrease in m6A occupancy of SETD2 was observed in METTL16‐depleted AGS cells (Figure 7C). Furthermore, the SRAMP online tool showed that nine high‐confidence potential m6A sites were within the CDS and 3'UTR of SETD2 mRNA, as depicted in Figure 7D. The MeRIP‐qPCR assay was performed to determine the enrichment of m6A in SETD2. The analysis revealed a notable reduction in m6A at a specific site (position 2*) within the CDS of SETD2 in both METTL16‐knockdown AGS and MKN‐28 cells (Figure 7E). Additionally, dual‐luciferase reporter assays showed that METTL16 knockdown resulted in a substantial decrease in the relative luciferase activity of the wild‐type SETD2 CDS, while it did not impact the relative luciferase activity of the mutant SETD2 CDS (Figure 7F). These findings indicate that METTL16 regulates the expression of SETD2 by methylating the m6A site in the CDS of SETD2 mRNA in GC cells.

## 4 Discussion

Gastric cancer (GC) is one of the common causes of cancer‐related deaths worldwide [28]. Notably, the poor prognosis of patients with this particular cancer type underscores the necessity for more research into the underlying mechanisms and the discovery of potential biomarkers. Here, by integrating microarray, TCGA RNA‐seq, and survival analysis data, the m6A methyltransferase METTL16 was identified as a potential oncogenic factor in GC.

METTL16, an m6A methyltransferase discovered recently, operates independently from the METTL3/14 complex and holds significance in a range of cancers, including hepatocellular carcinoma [29], pancreatic ductal adenocarcinoma [30], epithelial ovarian cancer [31], breast cancer [25], and cholangiocarcinoma [32]. Moreover, METTL16 was highly expressed in GC cells and tissues and promotes GC cell proliferation [33]. Consistent with these results, our study revealed significant upregulation of METTL16 in GC tissues and cells, and silencing METTL16 was found to inhibit GC progression. Additionally, low MET:736.TL16 expression is beneficial in the prognosis of GC patients. Extensive research has been conducted on the role of METTL16 in regulating cancer progression through m6A modification of target genes, including U6 snRNA [34], MALAT1 [35], XIST [36], and RAB11B‐AS1 [24], and mRNA (MAT2A [37], VPS33B [37], FGFR4 [32], and GPX4 [25]). However, current research on the target genes of METTL16 in GC has mainly focused on cyclin D1 [33] and ferredoxin 1 (FDX1) [38]. Here, UBXN1, identified as a target gene of METTL16, was selected through the application of WGCNA and integrated multi‐omics data. A significant positive correlation was observed between METTL16 and the UBXN1 gene, and the knockdown of METTL16 resulted in decreased expression of UBXN1 in GC cells.

The UBXN1 gene encodes a protein known as UBX domain‐containing protein 1‐like, which serves as a regulator of ubiquitin [39], and facilitates the proteolytic degradation of a specific subset of proteins during polyubiquitination [39]. An increasing number of studies have identified UBXN1 as a negative regulator of NF‐κB signaling [23, 40]. For example, lncRNA PRADX activates the NF‐κB pathway by inhibiting UBXN1 expression, thereby promoting the occurrence of glioblastoma and colon adenocarcinoma [41]. Nonetheless, there are currently no reports available on the involvement of UBXN1 in GC. We observed significantly increased expression of UBXN1 in both GC tissues and cells, and high UBXN1 expression was significantly correlated with poor prognosis. Knockdown of UBXN1 suppressed the malignant traits of GC cells through activation of the NF‐κB pathway. Additionally, the downregulation of METTL16 led to a decrease in UBXN1 expression through methylation at the m6A site within the CD of UBXN1 mRNA in GC cells. Further research revealed that METTL16‐mediated m6A modification of UBXN1 mRNA promotes the progression of GC. This study confirmed for the first time the cancer‐promoting effect of m6A‐modified UBXN1 in cancer.

Current studies have demonstrated the interplay between m6A and histone modifications in various cell types, such as human hepatoma cells HepG2, embryonic stem cells, and human erythroleukemia cells [42, 43, 44, 45, 46]. The absence of METTL3 significantly attenuates the changing levels of chromatin accessibility at promoters and gene bodies, and METTL3 exerts a negative control on the histone modifications H3K4me3 and H3K36me3 [43]. In addition, Liu et al. reported the collaboration between the histone acetyltransferase p300 and the transcription factor YY1 in governing METTL16 gene expression through H3K27 acetylation in cholangiocarcinoma cells [32]. However, there is limited research on the synergistic relationship between m6A and histone modifications in GC. Interestingly, we found that a reduction in METTL16 led to elevated H3K36me3 levels at the promoter region of UBXN1 in GC cells. Additional investigations revealed that METTL16 knockdown significantly increased the protein expression of SETD2, the major H3K36me3 methyltransferase, and the increased expression of SETD2 in METTL16 knockdown GC cells attributed to reduced m6A levels in the CDS of SETD2 mRNA. These results suggest that METTL16 knockdown not only decreases UBXN1 expression through m6A modification but also inhibits UBXN1 expression by elevating H3K36me3 modification at the UBXN1 promoter (Figure 7G). Our study on the collaboration between METTL16‐mediated m6A modification and histone modifications in GC aims to deepen our comprehension of the interrelationship between these two types of modifications.

Our study demonstrated the crucial role of METTL16 in driving GC progression through linking m6A modification with histone modifications. By regulating the malignant phenotype of GC cells at transcriptional and post‐transcriptional levels, METTL16 presents novel possibilities for cancer diagnosis and targeted therapy.

## Author Contributions

**Kesong Shi:** conceptualization (equal), validation (equal), visualization (equal), writing – original draft (equal), writing – review and editing (equal). **Yani Chen:** conceptualization (equal), validation (equal), visualization (equal), writing – original draft (equal), writing – review and editing (equal). **Tian Gao:** conceptualization (equal), validation (equal), visualization (equal), writing – original draft (equal), writing – review and editing (equal). **Hua Guo:** data curation (equal), formal analysis (equal), resources (equal), software (equal). **Xinyao Fu:** data curation (equal), formal analysis (equal), resources (equal), software (equal). **Yuan Wu:** investigation (equal), methodology (equal), visualization (equal). **Haiquan Yu:** conceptualization (equal), funding acquisition (equal), supervision (equal), writing – original draft (equal), writing – review and editing (equal).

## Ethics Statement

Approval of the research protocol by an Institutional Review Board: The study of human sample was approved by the Ethics Committee of Shanghai Outdo Biotech Company, Shanghai, China.

Informed Consent: Informed consent was obtained from all patients in accordance with a protocol approved by the ethics review board of Shanghai Outdo Biotech (approval no: YB M‐05‐02). Written informed consent was obtained from each subject.

Registry and the Registration No. of the study/trial: N/A.

Animal Studies: All experiments were approved by the Animal Care and Use Committee of Inner Mongolia University (approval ID: IMU‐2022‐mouse‐059). All animal experiments were conducted in specific pathogen‐free environments, in compliance with the guidelines for the Care and Use of Laboratory Animals.

## Conflicts of Interest

The authors declare no conflicts of interest.

## Supporting information


**Fig. S1.** **Overexpression of UBXN1 promoted the malignant phenotype of AGS and MKN‐28 cells in vitro A–B** qRT‐PCR and western blot analysis. **C–E** Proliferation of AGS and MKN‐28cells following UBXN1 overexpression. **F** Flow cytometry analysis of apoptosis in AGS and MKN‐28 cells. **G–H** Cell migratory and invasive capabilities were assessed in AGS and MKN‐28 cells. Bar = mean ± SD (*n* = 3). *p < 0.05, **p < 0.01, ***p < 0.001.

**Table S1.** The detailed information for each dataset. **Table S2.** The sequences of siRNAs. **Table S3.** Primer sequences of genes. **Table S4.** Antibody information. **Table S5.** Primer sequences of MeRIP‐qPCR. **Table S6.** The UBXN1 promoter sequences of primers. **Table S7.** Prognosis‐associated genes.

## Data Availability

The datasets employed in the current study are available from the corresponding author upon reasonable request.
