# Supplementary figures and images for: The Oncogenic Role of UBXN1 in Gastric Cancer Is Attributed to the METTL16‐Mediated m6A Methylation and Histone Modifications
Source: Cancer Med. 2025 Mar 17;14(6):e70772. doi: 10.1002/cam4.70772 (PMC11912429; doi:10.1002/cam4.70772)

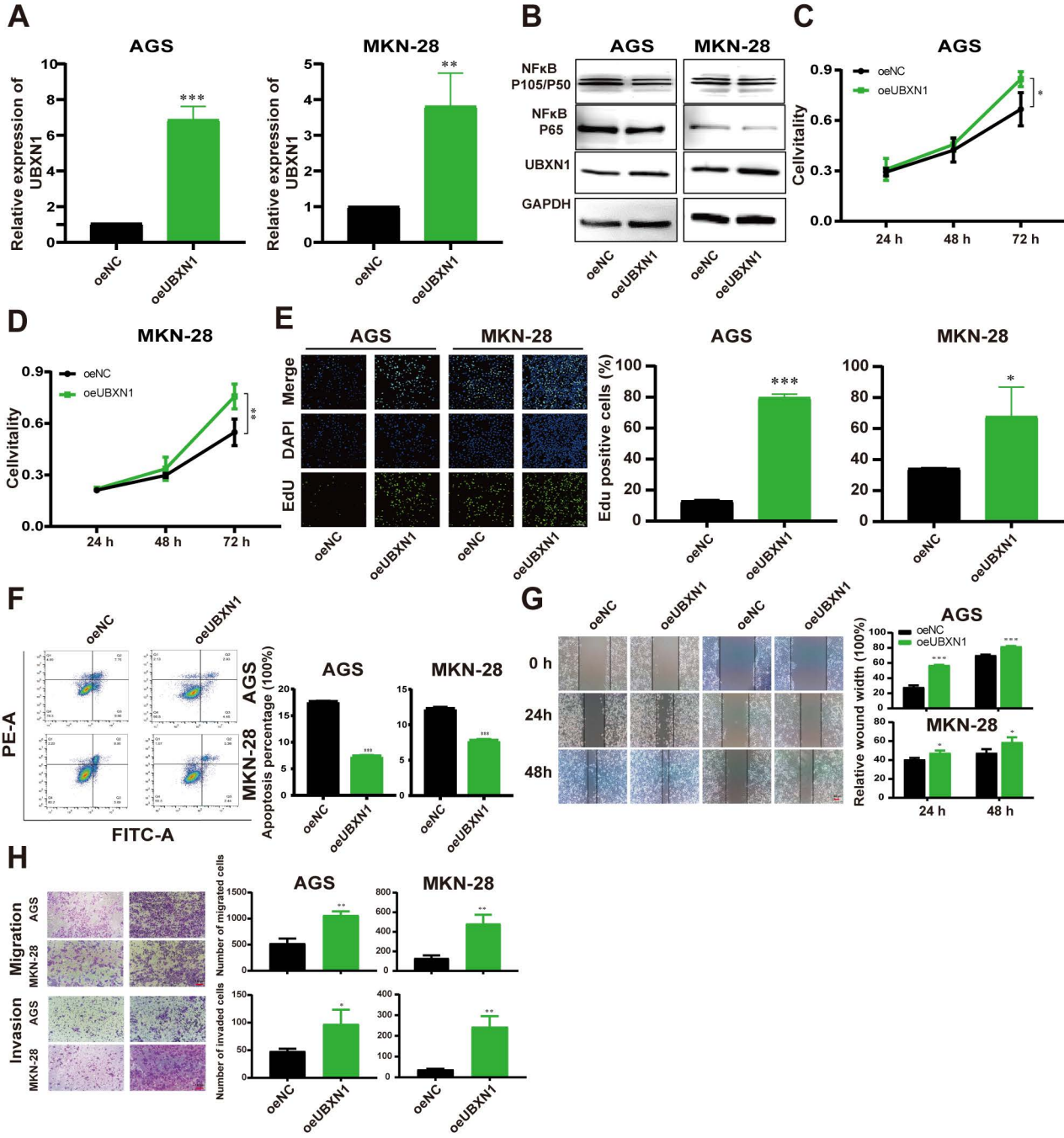

Supplement: Supplementary file 1 — Fig. S1. Overexpression of UBXN1 promoted the malignant phenotype of AGS and MKN‐28 cells in vitro A–B qRT‐PCR and western blot analysis. C–E Proliferation of AGS and MKN‐28cells following UBXN1 overexpression. F Flow cytometry analysis of apoptosis in AGS and MKN‐28 cells. G–H Cell migratory and invasive capabilities were assessed in AGS and MKN‐28 cells. Bar = mean ± SD (n = 3). *p < 0.05, **p < 0.01, ***p < 0.001. [file CAM4-14-e70772-s001.pdf]
