# Supplementary material for: The Oncogenic Role of UBXN1 in Gastric Cancer Is Attributed to the METTL16‐Mediated m6A Methylation and Histone Modifications
Source: Cancer Med. 2025 Mar 17;14(6):e70772. doi: 10.1002/cam4.70772 (PMC11912429; doi:10.1002/cam4.70772)
Supplement: Supplementary file 2 — Table S1. The detailed information for each dataset. Table S2. The sequences of siRNAs. Table S3. Primer sequences of genes. Table S4. Antibody information. Table S5. Primer sequences of MeRIP‐qPCR. Table S6. The UBXN1 promoter sequences of primers. Table S7. Prognosis‐associated genes. [file CAM4-14-e70772-s002.docx]

| GEO accession | Samples | Type |
| --- | --- | --- |
| GSE26899 | 12 Gastric Surrounding normal tissues;  96 Gastric tissues | microarray data |
| GSE186902 | 3 GES-1 cells | RNA-seq |
| GSE202165 | 4 MKN-28 cells | RNA-seq |
| GSE235631 | 3 AGS cells | RNA-seq |
| GSE224890 | 2 sh-METTL16;  2 sh-NC | MeRIP-seq |

**Table S1.** **The detailed information for each dataset**

**Table S2. The sequences of siRNAs**

| Target | Sequences |
| --- | --- |
| siRNA-control | S: UUCUCCGAACGUGUCACGUTT  AS: ACGUGACACGUUCGGAGAATT |
| siRNA-METTL16-1 | S: CCAUGACAGUCUACAACUUTT  AS: AAGUUGUAGACUGUCAUGGTT |
| siRNA -METTL16-2 | S: GGUCCAGCAUAAACGAGUUTT  AS: AACUCGUUUAUGCUGGACCTT |
| siRNA-UBXN1-1 | S: GCUGACGGCUCUUGAGAGUTT  AS: ACUCUCAAGAGCCGUCAGCTT |
| siRNA-UBXN1-2 | S: GGAGGCAUUGGAACGGGAATT  AS: UUCCCGUUCCAAUGCCUCCTT |

**Table S3. Primer sequences of genes**

| Genes | Sequences (5’-3’) |
| --- | --- |
| GAPDH | F: 5’- GCACCGTCAAGGCTGAGAAC -3’ |
|  | R:5’- TGGTGAAGACGCCAGTGGA -3’ |
| METTL16 | F: 5’- CTCTGACGTGTACTCTCCTAAGG -3’ |
|  | R: 5’- TACCAGCCATTCAAGGTTGCT -3’ |
| UBXN1 | F: 5’-GCCTAGGAAAAGGAGGGATG-3’ |
|  | R: 5’-GCCACAATGAGAACAGCAGA-3’ |

| Antibodies | Source | Identifier |
| --- | --- | --- |
| GAPDH | Proteintech,China | 60004-1-lg (1:1000) |
| UBXN1 | Proteintech,China | 16135-1-AP (1:1000) |
| SETD2 | Proteintech,China | 55377-1-AP (1:1000) |
| secondary antibodies | Proteintech,China | SA00001-1 or SA00001-2 （1:2000） |
| METTL16 | Abcam, Cambridge, UK | ab313743 (1:1000) |
| H3K9me3 | Abcam, Cambridge, UK | ab10812 (1:1000) |
| H3K4me3 | PTM Bio Inc., Hangzhou, China | PTM-5019 (1:1000) |
| H3K36me3 | PTM Bio Inc., Hangzhou, China | PTM-625RM (1:1000) |
| NFκB P65 | Servicebio,China | GB11997 (1:1000) |
| NFκB P105/50 | Servicebio,China | GB115431 (1:1000) |

**Table S4.** **Antibody information**

**Table S5.** **Primer sequences of MeRIP-qPCR**

| Genes | Sequences |
| --- | --- |
| UBXN1 | F: 5’- TCACAGGGAACCAGGGCATC-3’  R: 5’- TCTCCGGCAGCAGAACCAGA-3’ |
| 1# SETD2 | F: 5’- TACACAGATAACAGAGCACG-3’  R: 5’- TCATTAGGGGGAGAACAACA -3’ |
| 2# SETD2 | F: 5’- TAGTAGGTGCAAAGAAAAAG-3’  R: 5’- ATAACTGGCATAGACATGAG-3’ |
| 3# SETD2 | F: 5’- TAGATGCCACTCAAAAAGGA-3’  R: 5’- CTGGAACTGATAGTCAAACG-3’ |
| 4# SETD2 | F: 5’- ATCGCGTGCTCATACACCAC-3’  R: 5’- CTTTGCCATCCTTGCCTTCT-3’ |
| 5# SETD2 | F: 5’- CCTCCACCAGTACCAGTGGT-3’  R: 5’- TGTCTGTCCTTGATAATATA-3’ |
| 6# SETD2 | F: 5’-AGGATGGGTGGTCAGGTAAG-3’  R: 5’-GATGGAGTTCATTTTTGTGG-3’ |

**Table S6. The UBXN1 promoter sequences of primers**

| Gene | Sequences |
| --- | --- |
| 1#UBXN1 | F: 5’-TTTACTAAGTGAAGAGA-3’  R: 5’-CCTCCCAAAGTGCTAAG-3’ |
| 2#UBXN1 | F: 5’- GCGAGGCAGGTGGATCA-3’  R: 5’- GCGATTCTCCTGCCCGA-3’ |
| 3#UBXN1 | F: 5’- TTAAACCCGGGGCAGGC-3’  R: 5’- CTGGGATTACAGGCGTG-3’ |
| 4#UBXN1 | F: 5’-CACTTTGGGAGGCCGAG-3’  R: 5’-TCCTGGGTGCATGCGAT-3’ |
| 5#UBXN1 | F: 5’- GGCGGAGGTTGCAGTGA-3’  R: 5’- AATCATGTAGTCCTGTC-3’ |
| 6#UBXN1 | F: 5’-GTGTTTTGCAAAAATCC-3’  R: 5’-CGGAACTTCATCCTGCC-3’ |
| 7#UBXN1 | F: 5’-GGAACTGGCTTCATTCC-3’  R: 5’-GGGCGGGAAGGGTCAGC-3’ |

**Table S7. Prognosis-associated genes**

| Gene Logrank P Hazard Ratio  Hazard Ratio | | |
| --- | --- | --- |
| E2F1 | 1.80e-12 | 1.9 (1.59-2.28) |
| FBXO25 | 0.027 | 0.77 (0.62-0.97) |
| RANBP9 | 3.8e−03 | 0.76 (0.63-0.92) |
| UBXN1 | 6.80e-08 | 1.6 (1.35-1.9) |
|  |  |  |

**The code for the WGCNA**

if (!requireNamespace("BiocManager", quietly = TRUE))

install.packages("BiocManager")

BiocManager::install("impute")

BiocManager::install("WGCNA")

library("WGCNA")

library("impute")

options(stringsAsFactors=F)

collectGarbage()

rm(list=ls())

rt=read.table("A.txt",sep="\t",header=T,check.names=F)

rt=as.matrix(rt)

rownames(rt)=rt[,1]

exp=rt[,2:ncol(rt)]

dimnames=list(rownames(exp),colnames(exp))

exp=matrix(as.numeric(as.matrix(exp)),nrow=nrow(exp),dimnames=dimnames)

dim(exp)

length(colnames(exp))

datExpr0 = as.data.frame(t(exp))

gsg = goodSamplesGenes(datExpr0, verbose = 3)

gsg$allOK

if (!gsg$allOK){

if (sum(!gsg$goodGenes)>0)

printFlush(paste("Removing genes:", paste(names(datExpr0)[!gsg$goodGenes], collapse = ", ")));

if (sum(!gsg$goodSamples)>0)

printFlush(paste("Removing samples:", paste(rownames(datExpr0)[!gsg$goodSamples], collapse = ", ")));

datExpr0 = datExpr0[gsg$goodSamples, gsg$goodGenes]

}

sampleTree = hclust(dist(datExpr0), method = "average")

par(cex = 0.6)

par(mar = c(0,4,2,0))

plot(sampleTree)

plot(sampleTree, main = "Sample clustering to detect outliers", sub="", xlab="", cex.lab = 1.5, cex.axis = 1.5, cex.main = 2)

traitData=read.table("B_luan_traiData.txt",sep="\t",header=T,check.names=F)

traitData=as.matrix(traitData)

rownames(traitData)=traitData[,1]

exp2=traitData[,2:ncol(traitData)]

dimnames=list(rownames(exp2),colnames(exp2))

exp2=matrix(as.numeric(as.matrix(exp2)),nrow=nrow(exp2),dimnames=dimnames)

sameSample=intersect(rownames(datExpr0), rownames(exp2))

datExpr0=datExpr0[sameSample,]

datTraits=exp2[sameSample,]

sampleTree2 = hclust(dist(datExpr0), method = "average")

plot(sampleTree2)

traitColors = numbers2colors(datTraits, signed = FALSE)

traitColors = numbers2colors(datTraits, signed = FALSE)

plotDendroAndColors(sampleTree2, traitColors,

groupLabels = names(datTraits),

main = "Sample dendrogram and trait heatmap")

save(datExpr0, datTraits, file = "A2.RData")

rm(list = ls())

load(file = "A2.RData")

enableWGCNAThreads(10)

powers <- c(c(1:20), seq(from=12, to=20, by=2))

sft <- pickSoftThreshold(datExpr0, powerVector=powers, verbose=5, networkType="unsigned")

sft$powerEstimate

sizeGrWindow(6, 3)

par(mfrow=c(1, 2))

cex1 <- 0.9

plot(sft$fitIndices[, 1], -sign(sft$fitIndices[, 3])*sft$fitIndices[, 2],

xlab="Soft Threshold (power)", ylab="Scale Free Topology Model Fit,signed R^2", type="n",

main=paste("Scale independence"))

text(sft$fitIndices[, 1], -sign(sft$fitIndices[, 3])*sft$fitIndices[, 2],

labels=powers, cex=cex1, col="red")

abline(h=0.9, col="red")

plot(sft$fitIndices[, 1], sft$fitIndices[, 5],

xlab="Soft Threshold (power)", ylab="Mean Connectivity", type="n",

main=paste("Mean connectivity"))

text(sft$fitIndices[, 1], sft$fitIndices[, 5], labels=powers, cex=cex1, col="red")

sft$powerEstimate

sft$powerEstimate <-4

net <- blockwiseModules(datExpr0, power = sft$powerEstimate,

TOMType = "unsigned", minModuleSize = 30,

reassignThreshold = 0, mergeCutHeight = 0.25,

numericLabels = TRUE, pamRespectsDendro = FALSE,

saveTOMs = TRUE,

saveTOMFileBase = "femaleMouseTOM",

verbose = 3)

table(net$colors)

sizeGrWindow(12, 9)

mergedColors <- labels2colors(net$colors)

plotDendroAndColors(net$dendrograms[[1]], mergedColors[net$blockGenes[[1]]],

"Module colors",

dendroLabels = FALSE, hang = 0.03,

addGuide = TRUE, guideHang = 0.05)

moduleLabels <- net$colors

moduleColors <- labels2colors(net$colors)

MEs <- net$MEs

geneTree <- net$dendrograms[[1]]

save(MEs, moduleLabels, moduleColors, geneTree,

file="A3.RData")

load(file = "A3.RData")

TOM = TOMsimilarity(adjacency);

dissTOM = 1-TOM

geneTree = hclust(as.dist(dissTOM), method = "average");

sizeGrWindow(12,9)

plot(geneTree, xlab="", sub="", main = "Gene clustering on TOM-based dissimilarity",

labels = FALSE, hang = 0.04);

minModuleSize = 30;

dynamicMods = cutreeDynamic(dendro = geneTree, distM = dissTOM,

deepSplit = 2, pamRespectsDendro = FALSE,

minClusterSize = minModuleSize);

table(dynamicMods)

dynamicColors = labels2colors(dynamicMods)

table(dynamicColors)

sizeGrWindow(8,6)

plotDendroAndColors(geneTree, dynamicColors, "Dynamic Tree Cut",

dendroLabels = FALSE, hang = 0.03,

addGuide = TRUE, guideHang = 0.05,

main = "Gene dendrogram and module colors")

moduleColors = labels2colors(net$colors)

MEs = moduleEigengenes(datExpr0, moduleColors)$eigengenes

MET = orderMEs(MEs)

pdf(file="2eigengenes_trait_relationship.pdf",width=7, height=9)

par(cex = 0.9)

plotEigengeneNetworks(MET,"", marDendro=c(0,4,1,2),

marHeatmap=c(3,4,1,2), cex.lab=0.8, xLabelsAngle=90)

dev.off()

MEList = moduleEigengenes(datExpr0, colors = dynamicColors)

MEs = MEList$eigengenes

MEDiss = 1-cor(MEs);

METree = hclust(as.dist(MEDiss), method = "average");

sizeGrWindow(7, 6)

plot(METree, main = "Clustering of module eigengenes",

xlab = "", sub = "")

MEDissThres = 0.9

abline(h=MEDissThres, col = "red")

merge = mergeCloseModules(datExpr0, dynamicColors, cutHeight = MEDissThres, verbose = 3)

mergedColors = merge$colors;

mergedMEs = merge$newMEs;

pdf(file="5eigengenes_trait_relationship.pdf",width=7, height=9)

par(cex = 0.9)

plotEigengeneNetworks(mergedMEs,"", marDendro=c(0,4,1,2),

marHeatmap=c(3,4,1,2), cex.lab=0.8, xLabelsAngle=90)

dev.off()

sizeGrWindow(12, 9)

plotDendroAndColors(geneTree, cbind(dynamicColors, mergedColors),

c("Dynamic Tree Cut", "Merged dynamic"),

dendroLabels = FALSE, hang = 0.03,

addGuide = TRUE, guideHang = 0.05)

moduleColors = mergedColors

table(moduleColors)

colorOrder = c("grey", standardColors(50))

moduleLabels = match(moduleColors, colorOrder)-1

MEs = mergedMEs

nGenes = ncol(datExpr0)

nSamples = nrow(datExpr0)

moduleTraitCor = cor(MEs, datTraits, use = "p")

moduleTraitPvalue = corPvalueStudent(moduleTraitCor, nSamples)

textMatrix = paste(signif(moduleTraitCor, 2), "\n(",

signif(moduleTraitPvalue, 1), ")", sep = "")

dim(textMatrix) = dim(moduleTraitCor)

par(mar = c(5, 15, 2, 2))

labeledHeatmap(Matrix = moduleTraitCor,

xLabels = colnames(datTraits),

yLabels = names(MEs),

ySymbols = names(MEs),

colorLabels = FALSE,

colors = blueWhiteRed(50),

textMatrix = textMatrix,

setStdMargins = FALSE,

cex.text = 0.4,

zlim = c(-1,1),

main = paste("Module-trait relationships"))

dev.off()

modNames = substring(names(MEs), 3)

geneModuleMembership = as.data.frame(cor(datExpr0, MEs, use = "p"))

MMPvalue = as.data.frame(corPvalueStudent(as.matrix(geneModuleMembership), nSamples))

names(geneModuleMembership) = paste("MM", modNames, sep="")

names(MMPvalue) = paste("p.MM", modNames, sep="")

traitNames=names(datTraits)

geneTraitSignificance = as.data.frame(cor(datExpr0, datTraits, use = "p"))

GSPvalue = as.data.frame(corPvalueStudent(as.matrix(geneTraitSignificance), nSamples))

names(geneTraitSignificance) = paste("GS.", traitNames, sep="")

names(GSPvalue) = paste("p.GS.", traitNames, sep="")

for (trait in traitNames){

traitColumn=match(trait,traitNames)

for (module in modNames){

column = match(module, modNames)

moduleGenes = moduleColors==module

if (nrow(geneModuleMembership[moduleGenes,]) > 1){

outPdf=paste(trait, "_", module,".pdf",sep="")

pdf(file=outPdf,width=7,height=7)

par(mfrow = c(1,1))

verboseScatterplot(abs(geneModuleMembership[moduleGenes, column]),

abs(geneTraitSignificance[moduleGenes, traitColumn]),

xlab = paste("Module Membership in", module, "module"),

ylab = paste("Gene significance for ",trait),

main = paste("Module membership vs. gene significance\n"),

cex.main = 1.2, cex.lab = 1.2, cex.axis = 1.2, col = module)

abline(v=0.8,h=0.5,col="red")

dev.off()

}

}

}

module = "blue"

column = match(module, modNames);

moduleGenes = moduleColors==module;

sizeGrWindow(7, 7);

par(mfrow = c(1,1));

verboseScatterplot(abs(geneModuleMembership[moduleGenes, column]),

abs(geneTraitSignificance[moduleGenes, 1]),

xlab = paste("Module Membership in", module, "module"),

ylab = "Gene significance for CB",

main = paste("Module membership vs. gene significance\n"),

cex.main = 1.2, cex.lab = 1.2, cex.axis = 1.2, col = module)

for (mod in 1:nrow(table(moduleColors)))

{

modules = names(table(moduleColors))[mod]

probes = colnames(datExpr0)

inModule = (moduleColors == modules)

modGenes = probes[inModule]

write.table(modGenes, file =paste0(modules,".txt"),sep="\t",row.names=F,col.names=F,quote=F)

}

set.seed(10);

select = sample(nGenes, size = nSelect);

selectTOM = dissTOM[select, select];

selectTree = hclust(as.dist(selectTOM), method = "average")

selectColors = mergedColors[select];

sizeGrWindow(9,9)

plotDiss = selectTOM^7;

diag(plotDiss) = NA;

TOMplot(plotDiss, selectTree, selectColors, main = "Network heatmap plot, selected genes")

TOMplot(plotDiss, selectTree, selectColors, main = "Network heatmap plot, all genes", col=gplots::colorpanel(250,'red',"orange",'lemonchiffon'))
